# Supplementary material for: Computing the effects of excitatory-inhibitory balance on neuronal input-output properties
Source: PLoS Comput Biol. 2026 Mar 9;22(3):e1013958. doi: 10.1371/journal.pcbi.1013958 (PMC12998957; doi:10.1371/journal.pcbi.1013958)
Supplement: S1 Appendix — (PDF) [file pcbi.1013958.s005.pdf]

Appendix

Model and parameters

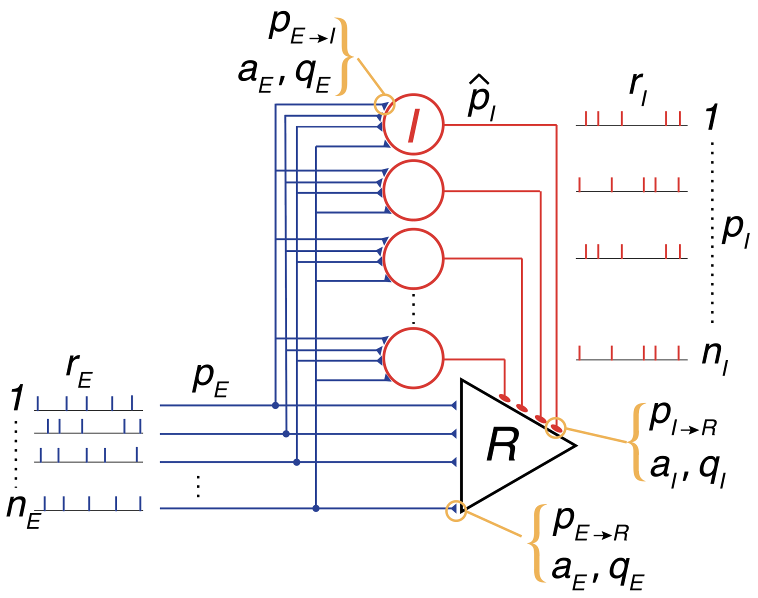

Fig S1. Model parameters.

The network is configured as a feedforward inhibitory circuit, with a reference neuron (triangle, Fig. S1) and n_I inhibitory neurons, each receiving n_E excitatory inputs from an external source; the reference neuron also receives inputs from the local inhibitory neurons and is the main output of the network. In response to the stimulus, the external afferents fire either a single (brief) action potential or trains (sustained) of action potentials. The model parameters are defined as follows.

<u>**Afferents:**</u>

n_E : number of afferents (100–250)

p_E : probability that an afferent fires in response to a stimulus

r_E : firing rate of afferents for long-duration stimuli (set to 50 Hz)

$p_{E \to R}$: probability that an afferent spike evokes an EPSP in the reference cell (synaptic efficacy). For simplicity, this is set to 1, indicating a reliable synapse.

$p_{E \to I}$: probability that an afferent spike evokes an EPSP in an inhibitory cell ($p_{E \to I} = 1$ for a reliable synapse).

a_E : amplitude of a single excitatory synaptic potential (250 μV) or current (10.3 pA) in both I cells and the reference cell.

a_I : amplitude of a single inhibitory synaptic potential (-250 μV) or current (-10.3 pA) in the reference cell.

q_E : charge transfer of a single excitatory synapse in both I cells and the reference cell (0.0557 pC).

q_I : charge transfer (-0.0557 pC) of a single inhibitory synapse on the reference cell, expressed as a fraction of excitatory charge transfer:

$$q_I = k_q q_E, \quad k_q \in [0, 1].$$

n_I : number of inhibitory neurons (20–50), also expressed as a fraction of the number of excitatory afferents:

$$n_I = k_n n_E.$$

$\hat{p}_I$: probability that an inhibitory neuron fires during a stimulus

r_I : firing rate of inhibitory neurons, also expressed as

$$r_I = k_r r_E, \quad k_r \geq 0.$$

$p_{I\to R}$: probability that a spike from an inhibitory neuron evokes an IPSP in the reference cell ($p_{I\to R} = 1$ for a reliable synapse). Also expressed relative to excitatory synaptic efficacy:

$$k_{EI} = \frac{p_{I\to R}}{p_{E\to R}}.$$

To simplify simulations:

1. $p_{E\to R}$ is set to 1 so that the probability of an EPSP appearing in the reference cell is simply p_E.

2. The effective probability p_I that an IPSP appears in the reference cell—accounting for the number, strength, and firing rate of inhibitory inputs relative to excitatory inputs—is defined as

$$p_I = \hat{p}_I \, k_n \, k_q \, k_r \, k_{EI} \in [0, 1]. \tag{1}$$

Calculating firing probability with sustained input

Many neurons, in response to sustained synaptic input, enter the oscillatory firing regime. Typically, the firing rate depends on the mean synaptic current (the so-called firing rate–current, or f–I, curve). During a sustained stimulus, the afferents and I neurons generate trains of excitatory and inhibitory synaptic inputs, respectively, producing a synaptic barrage in the postsynaptic neuron.

The first step is to express the excitatory and inhibitory synaptic currents, and hence the net synaptic current, in terms of p_E and p_I. During a stimulus, each afferent is modeled as generating a Poisson spike train,

$$s^E_{\mathrm{spk}}(t) = \sum_i \delta(t - t_i), \tag{S1}$$

where $\{t_i\}$ are the event times of a homogeneous Poisson process with rate λ_E.

The excitatory postsynaptic current (EPSC) train generated by a single afferent is given by

$$i^{(j)}_{E\to R}(t) = a_E\,(s^{E,j}_{\mathrm{spk}} * u_{\mathrm{PSC}})(t) = a_E \sum_i u_{\mathrm{PSC}}(t - t^{(j)}_i), \tag{S2}$$

where $*$ denotes convolution. The kernel $u_{\mathrm{PSC}}(t)$ represents the unitary EPSC generated by a single afferent and is normalized to unit peak, so that the amplitude a_E specifies the EPSC peak magnitude.

If there are n_E afferents, the total excitatory current is

$$i_{E\to R}(t) = a_E \sum_{j=1}^{n_E} \sum_k u_{\mathrm{PSC}}\left(t - t^{(j)}_k\right), \tag{S3}$$

where $t^{(j)}_k$ denotes the k-th spike time of afferent j.

If a stimulus causes each of the n_E excitatory afferents to fire with probability p_E, and if each spike successfully generates an EPSC in the reference cell with probability $p_{E\to R}$, then the effective rate of excitatory events is

$$\lambda_E = p_E\, p_{E\to R}\, n_E\, r_E. \tag{S4}$$

The corresponding mean excitatory current is therefore

$$\bar{i}_{E\to R} = p_E\, p_{E\to R}\, n_E\, r_E\, q_E, \qquad q_E = a_E \int_{-\infty}^{+\infty} u_{\mathrm{PSC}}(t)\, dt, \tag{S5}$$

where q_E denotes the total charge transfer associated with a single EPSC, and a_E is a scaling factor converting the normalized postsynaptic current waveform $u_{\mathrm{PSC}}(t)$ to physical units.

To calculate the inhibitory current into the reference cell during stimulation, the spike trains of the I neurons are first considered. The afferent inputs to the I neurons are analogous to those to the reference cell and yield the mean excitatory current

$$\bar{i}_{E\to I} = p_E\, p_{E\to I}\, n_E\, r_E\, q_E, \tag{S6}$$

where $p_{E\to I}$ denotes the probability that an afferent spike evokes an EPSP in an I neuron. When this mean excitatory current exceeds the rheobase current i_{rh}, each I neuron enter the oscillatory firing regime and generate spike trains

$$s^I_{\mathrm{spk}}(t) = \sum_k \delta(t - t_k), \tag{S7}$$

with firing rate r_I, which depends on the input current and need not follow a Poisson process.

The inhibitory current generated in the reference cell by the population of inhibitory neurons is computed in several stages. First, the probability that an inhibitory neuron fires in response to a stimulus is defined as

$$\hat{p}_I = \Pr(\text{I cell fires} \mid \text{stim}). \tag{S8}$$

For simulations with an LIF model, $\hat{p}_I$ was empirically measured as the probability that a stimulus evoked at least one spike during the stimulus interval (0.5–1 s). In principle, $\hat{p}_I$ can also be expressed analytically as the probability that an inhibitory cell receives at least a threshold number of excitatory inputs within an integration time window.

If there are n_I inhibitory neurons firing asynchronously, and each spike generates an IPSC in the reference cell with probability $p_{I\to R}$, then the total inhibitory current is

$$i_{I\to R}(t) = \hat{p}_I\, p_{I\to R}\, q_I \sum_{j=1}^{n_I} \sum_k \delta\big(t - t_k^{(j)}\big), \qquad q_I = a_I \int_{-\infty}^{+\infty} u_{\mathrm{PSC}}(t)\, dt, \tag{S9}$$

where q_I denotes the charge transfer associated with a single IPSC.

The mean inhibitory current is given by

$$\begin{aligned} \bar{i}_{I\to R} &= \hat{p}_I\, p_{I\to R}\, n_I\, q_I\, r_I \\ &= p_I\, p_{E\to R}\, n_E\, q_E\, r_E, \end{aligned} \tag{S10}$$

where p_I is the effective probability that an inhibitory input reaches the reference cell and accounts for network size, synaptic efficacy, synaptic strength, and relative firing rate at the reference cell:

$$p_I = \hat{p}_I\, k_n\, k_q\, k_r\, k_{EI}, \tag{S11}$$

with $k_n = \frac{n_I}{n_E}$ denoting the ratio of neuron numbers, $k_q = |\frac{q_I}{q_E}|$ the ratio of charge transfers, $k_r = \frac{r_I}{r_E}$ the ratio of firing rates, and $k_{EI} = \frac{p_{I\to R}}{p_{E\to R}}$ the ratio of synaptic efficacies.

Because of the feed-forward structure, the surviving excitatory current is

$$\begin{aligned} \bar{i}_{\mathrm{net}} &= p_E\, p_{E\to R}\, n_E\, r_E\, q_E - p_E\, p_I\, p_{E\to R}\, n_E\, r_E\, q_E \\ &= p_{E\to R}\, n_E\, r_E\, q_E \Big[p_E - p_E p_I\Big] \\ &= p_{E\to R}\, n_E\, r_E\, q_E\, p_E\big(1 - p_I\big) \\ &= p_{E\to R}\, n_E\, r_E\, q_E\, p_{\mathrm{net}}, \end{aligned} \tag{S12}$$

where the effective net excitation probability is defined as

$$p_{\mathrm{net}} = p_E\,(1 - p_I). \tag{S13}$$

This expression represents the probability that an EPSP in the reference cell survives feed-forward inhibition recruited by the same presynaptic excitatory population.

In the sub-oscillatory (subthreshold) regime, firing may still occur due to voltage fluctuations that produce random threshold crossings. Let X denote the number of *surviving* (uncanceled) EPSPs received during the interval $[t_0, t_0 + \Delta t]$, so that $X \sim \mathrm{Binom}(n_E, p_{\mathrm{net}})$, where $p_{\mathrm{net}} = p_E\,(1 - p_I)$. The fluctuation-driven firing probability is then

$$\begin{aligned} \Pr(X \ge n_\theta) &= 1 - \Pr(X < n_\theta) \\ &= 1 - \mathrm{BinomCDF}\big(n_\theta - 1;\, n_E,\, p_{\mathrm{net}}\big), \end{aligned} \tag{S14}$$

where n_E is the number of presynaptic excitatory afferents and n_θ is the minimum number of *uncanceled* EPSPs required to reach rheobase within Δt.

The probability above is sigmoidal in p_{net} and approaches unity as the input approaches the oscillatory (tonic spiking) regime. To obtain a continuous transition between fluctuation-driven and oscillatory firing, define

$$r_f(\bar{i}_{\mathrm{net}}) = \begin{cases} \Pr(X \ge n_\theta)\, r_{rh}, & \text{if } \bar{i}_{\mathrm{net}} \le i_{rh}, \\ r_{\mathrm{osc}}(\bar{i}_{\mathrm{net}}), & \text{if } \bar{i}_{\mathrm{net}} > i_{rh}, \end{cases} \tag{S15}$$

where

$$r_{\text{osc}}(\bar{i}_{\text{net}}) = \left[\tau_m \ln\left(\frac{\bar{i}_{\text{net}}}{\bar{i}_{\text{net}} - i_{rh}} \right) \right]^{-1}. \tag{S16}$$

An alternative interpretation, under a Poisson point-process view, is that at rheobase the probability of at least one spike in a window of duration Δt is

$$p_{\text{spike}}(\Delta t) = 1 - e^{-r_{rh}\,\Delta t} \;\approx\; r_{rh}\,\Delta t = \frac{\Delta t}{T}, \tag{S17}$$

where T is the mean interspike interval at rheobase ($r_{rh} = 1/T$) and the approximation holds for $\Delta t \ll T$. Fluctuation-driven firing then scales this per-bin probability by the survival tail:

$$p_{\text{spike}}^{\text{fluct}}(\Delta t) \;\approx\; \Pr\big(X \geq n_\theta\big)\,\frac{\Delta t}{T}, \tag{S18}$$

with $X \sim \text{Binom}(n_E, p_{\text{net}})$ as defined above. Equivalently, dividing by Δt yields the subthreshold rate

$$r_f \;=\; \Pr(X \geq n_\theta)\, r_{rh}. \tag{S19}$$

Calculating firing probability with transient input

In contrast to sustained stimulation, brief inputs produce a near-synchronous arrival of EPSPs and IPSPs, placing the neuron in a regime where spiking is highly sensitive to both the amplitude and precise timing of synaptic events. Consequently, the derivation and resulting expressions differ from those obtained under sustained input.

The analysis considers a reference neuron that receives n_E excitatory inputs from an external source and n_I inhibitory inputs from local interneurons. During stimulation, afferent EPSPs appear in both the reference cell and the local interneurons with probability p_E. The probabilities that an afferent spike evokes an EPSP in the reference and I cells are denoted by $p_{E\to R}$ and $p_{E\to I}$, respectively. Thus, the probabilities of observing an EPSP in each target cell are

$$\begin{aligned} \Pr(\text{EPSP in R} \mid \text{stim}) &= p_E p_{E\to R}, \\ \Pr(\text{EPSP in I} \mid \text{stim}) &= p_E p_{E\to I}. \end{aligned} \tag{S20}$$

If each afferent fires a single action potential whose timing is Gaussian distributed during the stimulus, the expected compound EPSP evoked in the reference cell is given by

$$\begin{aligned} C_E(t) &= a_E n_E p_E p_{E\to R} \int_{-\infty}^{\infty} \phi_E(\tau; \mu_t, \sigma_t^2) u_{\text{PSP}}(t-\tau)\, d\tau \\ &= a_E n_E p_E p_{E\to R}\, \tilde{p}_E(t), \end{aligned} \tag{S21}$$

where $\phi_E(t; \mu_t, \sigma_t^2)$ is the Gaussian probability density of presynaptic spike times centered at μ_t with temporal dispersion σ_t. The kernel $u_{\text{PSP}}(t)$ describes the time course of a unitary postsynaptic potential (e.g., an alpha function) with unit peak amplitude, a_E is the amplitude (in mV) of a single EPSP, and n_E is the number of afferents. The quantity $p_E p_{E\to R} \tilde{p}_E(t)$ therefore describes the time-dependent profile of excitatory input arriving at the reference cell.

The arrival-time density of inhibition in the reference cell can be empirically estimated by constructing a per-trial histogram $h_I(t)$ of inhibitory spike times pooled across the n_I inhibitory neurons and normalized by n_I:

$$\mathcal{P}_I(t) = \frac{h_I(t)}{n_I}. \tag{S22}$$

Here $h_I(t)$ denotes a histogram normalized by bin width, so that $\mathcal{P}_I(t)$ is a probability density.

To isolate the timing of inhibition specifically driven by the stimulus, a conditional arrival-time density is computed using only stimulus presentations that successfully elicit inhibitory spiking. A histogram $h_I(t \mid \text{stim})$ of inhibitory spike times is constructed from these responsive trials and normalized by both the number of inhibitory neurons n_I and the number of such stimulus repetitions n_{stim}:

$$\mathcal{P}_I(t \mid \text{stim}) = \frac{h_I(t \mid \text{stim})}{n_I\, n_{\text{stim}}}, \qquad \int_{-\infty}^{\infty} \mathcal{P}_I(t \mid \text{stim})\, dt = 1. \tag{S23}$$

This conditional density therefore represents the temporal distribution of inhibitory activity given that the stimulus successfully activated the inhibitory population.

The time-varying inhibitory profile in the reference cell during stimulation is given by

$$\tilde{p}_I(t \mid \text{stim}) = \int_{-\infty}^{\infty} \mathcal{P}_I(\tau \mid \text{stim}) u_{\text{PSP}}(t-\tau)\, d\tau, \tag{S24}$$

where $u_{\text{PSP}}(t)$ denotes the unitary postsynaptic potential waveform normalized to unit peak.

The effective inhibitory probability p_I is defined as

$$p_I := k_n k_a k_{EI}, \qquad p_I \in [0,1], \tag{S25}$$

where $k_n = n_I/n_E$ is the inhibitory-to-excitatory input ratio, $k_a = |a_I/a_E|$ is the ratio of inhibitory to excitatory synaptic amplitudes, and $k_{EI} = p_{I\to R}/p_{E\to R}$ is the ratio of synaptic efficacies. Unlike the sustained-stimulus case (Eq. S11), this expression does not include $\hat{p}_I$, which is conditioned out by restricting the analysis to stimulus-evoked inhibitory activity, nor k_r, since firing rate is irrelevant for single-spike responses.

The compound inhibitory postsynaptic potential can then be expressed relative to the compound excitatory potential as

$$C_I(t) = a_E n_E p_{E\to R} p_I \tilde{p}_I(t \mid \text{stim}), \tag{S26}$$

where $\tilde{p}_I(t \mid \text{stim})$ captures the temporal profile of inhibition and p_I determines its overall magnitude.

The net compound synaptic drive is therefore

$$\begin{aligned} C_{\text{net}}(t) &= C_E(t) - C_I(t) \\ &= a_E n_E p_{E\to R}[p_E \tilde{p}_E(t) - p_I \tilde{p}_I(t \mid \text{stim})] \\ &= a_E n_E p_{E\to R} \tilde{p}_{\text{net}}(t), \end{aligned} \tag{S27}$$

where $\tilde{p}_{\text{net}}(t)$ represents the effective time-dependent profile of net excitation.

Because the difference $p_E \tilde{p}_E(t) - p_I \tilde{p}_I(t \mid \text{stim})$ can become negative (for example when inhibition peaks after the falling phase of excitation), a lower bound of zero is imposed so that $\tilde{p}_{\text{net}}(t) \geq 0$.

Moreover, because inhibition in a feedforward circuit co-varies with excitation and both vanish in the absence of a stimulus, the net excitation can be written as

$$\tilde{p}_{\text{net}}(t) = p_E \tilde{p}_E(t) - p_I \tilde{p}_I(t \mid \text{stim}).$$

When excitation is present ($p_E \tilde{p}_E(t) > 0$), this expression can be equivalently rewritten as

$$\tilde{p}_{\text{net}}(t) = p_E \tilde{p}_E(t) \left[1 - \frac{p_I \tilde{p}_I(t \mid \text{stim})}{p_E \tilde{p}_E(t)}\right], \tag{S28}$$

where the ratio

$$\frac{p_I \tilde{p}_I(t \mid \text{stim})}{p_E \tilde{p}_E(t)}$$

quantifies the instantaneous relative strength of inhibition to excitation.

The probability that the cell fires at time t is the probability that the membrane potential exceeds the threshold v_θ. Equivalently, this is the probability that the number of excitatory postsynaptic potentials surviving inhibition exceeds the minimum number n_θ required to reach threshold. Let $X(t)$ denote the number of uncanceled EPSPs received during the interval $[t, t+\Delta t]$. The firing probability is then given by the upper tail of a binomial distribution:

$$\begin{aligned} p_f(t) &= \Pr\big(X(t) \geq n_\theta\big) \\ &= 1 - \beta(n_E, n_\theta, \tilde{p}_{\text{net}}(t)), \end{aligned} \tag{S29}$$

where $\beta(N,k,p) = \Pr(X < k) = \sum_{j=0}^{k-1} \binom{N}{j} p^j (1-p)^{N-j}$ is the cumulative distribution function of the binomial distribution with N trials and success probability p. The green curve in Fig. 6A (bottom panel, main text) illustrates this firing probability.

Finally, the probability that an action potential occurs at a specific time t_i is given by the probability of firing at t_i multiplied by the probability of not having fired at any earlier time:

$$\Pr(\text{spike at } t_i) = p_f(t_i) \prod_{j=1}^{i-1} [1 - p_f(t_j)]. \tag{S30}$$

This expression corresponds to the magenta curve in Fig. 6A (middle panel).

Effects on gain control

For transient stimulation, the instantaneous probability density of net excitation is given by Eq. S28,

$$\tilde{p}_{\text{net}}(t) = p_E\, \tilde{p}_E(t) \left[1 - \frac{p_I\, \tilde{p}_I(t \mid \text{stim})}{p_E\, \tilde{p}_E(t)}\right],$$

where the bracketed term describes the instantaneous balance between inhibition and excitation. This equation provides a compact description of how excitatory and inhibitory inputs combine at the level of synaptic drive; however, firing probability is determined by the interaction of this transient drive with temporal integration, thresholding, and trial-to-trial variability. Consequently, changes in the balance term do not map directly onto changes in the slope of the input–output (I–O) relation.

For transient inputs, firing is dominated by a brief temporal window near the rising phase of the excitatory drive, during which the net input first exceeds threshold. Mechanistically, any manipulation that delays threshold crossing without altering the local sensitivity of the net drive to changes in p_E will primarily shift the I–O curve along the input axis, rather than change its slope.

Case (i): inhibition independent of excitation (Fig. 6B*i*). When inhibition is independent of excitation, p_I is fixed while p_E varies. In this case, increasing inhibition subtracts an approximately constant contribution from the net drive during the spike-relevant window, delaying the time at which threshold is crossed. Because the local sensitivity of the net drive to changes in p_E at threshold crossing is largely unaffected, this delay manifests as a horizontal shift of the I–O relation without altering its slope.

Case (ii): inhibition proportional to excitation (Fig. 6B*ii*). When inhibition scales proportionally with excitation, implemented by setting $p_I \propto p_E$, the instantaneous balance between excitation and inhibition changes, but over the spike-relevant portion of the transient the fraction of excitation canceled remains approximately constant. As in Case (i), increasing inhibition primarily delays threshold crossing without substantially altering the sensitivity of firing probability to further increases in p_E. As a result, the I–O curves again exhibit a threshold shift with preserved slope.

Case (iii): inhibition recruited through feedforward excitation (Fig. 6B*iii*). In contrast, when inhibition is recruited through feedforward excitation, changes in p_E alter not only the timing of threshold crossing but also the sensitivity of threshold crossing to further increases in excitatory drive. Because inhibitory recruitment grows with p_E during the same temporal window that determines spike initiation, inhibition does not act as a fixed offset or simple rescaling of the net drive. Instead, it reduces the incremental increase in surviving excitation produced by additional increases in p_E.

Correcting for conductance

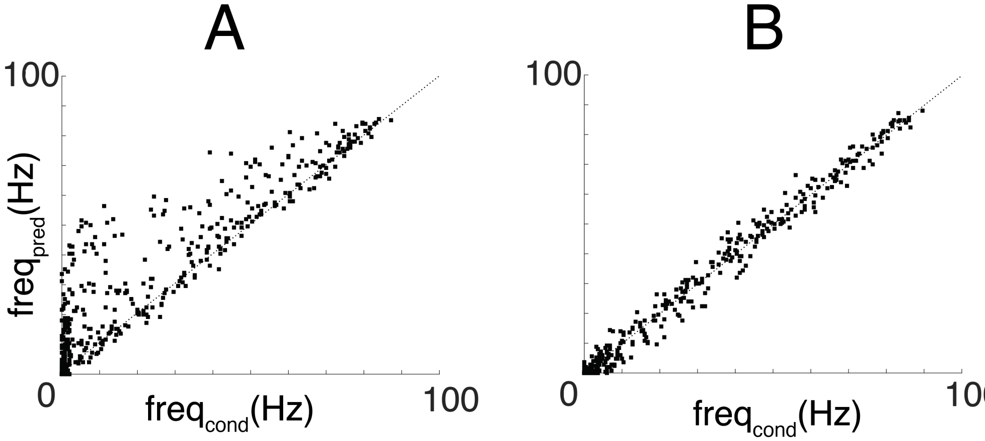

Fig S2. Effects of conductance. **A**, plot of firing rate predicted with EPSPs with probability p_{net} vs firing rate evoked with conductance-based excitatory and inhibitory EPSPs and IPSPs. **B**, same but with predicted frequency corrected for voltage-dependent non-linear summation of PSPs.

The voltage-dependent unitary postsynaptic currents for excitation and inhibition (in nA) were defined as

$$\mu_E(v,t) = g_E(t)\,(v - V_{\text{exc}}), \qquad \mu_I(v,t) = g_I(t)\,(v - V_{\text{inh}}), \tag{S31}$$

with corresponding charge transfers

$$q_E(v) = \int_{-\infty}^{\infty} \mu_E(v,t)\,dt = \left(\int_{-\infty}^{\infty} g_E(t)\,dt\right)(v - V_{\text{exc}}), \tag{S32}$$
$$q_I(v) = \int_{-\infty}^{\infty} \mu_I(v,t)\,dt = \left(\int_{-\infty}^{\infty} g_I(t)\,dt\right)(v - V_{\text{inh}}).$$

Here, the charge transfer $q(v)$ has units of picoCoulombs (pC), and the conductances $g_E(t)$ and $g_I(t)$ have units of nanoSiemens (nS). Each conductance followed an alpha

function:

$$g_E(t) = g_{E,\max}\,\frac{t}{\tau}\,e^{(1-t/\tau)}, \qquad \tau = 2\text{ ms}, \tag{S33}$$
$$g_I(t) = g_{I,\max}\,\frac{t}{\tau}\,e^{(1-t/\tau)}.$$

The mean voltage-dependent excitatory and inhibitory currents are

$$\begin{aligned}\bar{i}_E(\bar{v}) &= p_E\,p_{E\to R}\,r_E\,n_E\,q_E(\bar{v}),\\ \bar{i}_I(\bar{v}) &= \hat{p}_I\,p_{I\to R}\,r_I\,n_I\,q_I(\bar{v})\\ &= p_I\,p_{E\to R}\,r_E\,n_E\,q_E(\bar{v}),\end{aligned} \tag{S34}$$

where $\bar{v}$ denotes the mean membrane potential under synaptic input in the absence of spiking, $\hat{p}_I$ is the probability that a stimulus evokes firing in an I cell (see above), and p_I is the effective inhibitory probability given by

$$p_I = \hat{p}_I\,k_n\,k_{EI}\,k_r\,k_{qv}(\bar{v}), \tag{S35}$$

with

$$k_n = \frac{n_I}{n_E}, \qquad k_r = \frac{r_I}{r_E}, \qquad k_{EI} = \frac{p_{I\to R}}{p_{E\to R}}, \qquad k_{qv}(\bar{v}) = -\frac{q_I(\bar{v})}{q_E(\bar{v})}. \tag{S36}$$

Thus, the net current is given by

$$\begin{aligned}\bar{i}_{\text{net}}(\bar{v}) &= \bar{i}_E(\bar{v}) - p_E\,\bar{i}_I(\bar{v})\\ &= r_E\,n_E\,q_E(\bar{v})\,p_{E\to R}\,p_E\,[1-p_I]\\ &= r_E\,n_E\,q_E(\bar{v})\,p_{E\to R}\,p_{\text{net}}.\end{aligned} \tag{S37}$$

To examine how nonlinear summation affects predicted firing, the simulations described in the main text (Methods) were repeated with synaptic inputs modeled as conductances rather than currents. To produce equal PSP amplitudes (+250 μV for EPSPs and −250 μV for IPSPs) at the resting potential (−70 mV), with excitatory and inhibitory reversal potentials of 0 and −80 mV, respectively, the maximum inhibitory conductance ($g_{I,\max} = 1.045$ nS) was set to be seven times larger than the maximum excitatory conductance ($g_{E,\max} = 0.147$ nS). The firing rate in this conductance-based simulation mode is denoted f_{cond}.

The predicted firing rate f_{pred} was obtained as follows. First, the mean evoked membrane potential $\bar{v}$ was estimated by repeating the simulations above with action potentials disabled. Second, a Poisson spike train was generated with rate $\lambda = n_E p_{\text{net}} r_E$ (with $p_{E\to R} = 1$). Third, the net synaptic current was obtained by convolving this spike train with a peak-normalized alpha function scaled by the excitatory conductance amplitude (a_E; see Methods). Finally, the resulting current was delivered to the LIF neuron, and the firing rate was measured.

The firing of the reference cell in response to input depends on the parameters listed above (Model and Parameters) and in Table 2 of the Methods, which are summarized compactly in Eq. S37. To evaluate the effectiveness of the conductance correction expression (Eq. S37), simulations were performed using random values for selected parameters (p_E, $p_{E\to I}$, and k_n), while keeping the remaining parameters fixed. As noted in the main text, changes in $p_{E\to I}$ affected the firing rate r_I (and hence k_r) of inhibitory neurons and altered the resulting I–O curves. Similarly, variations in k_n influenced the magnitude of inhibition to the reference cell and its I–O relationship. Accordingly, 1000 trials were run with random combinations of p_E (0.6–1; 0.6 being the minimum required to evoke firing), $p_{E\to I}$ (0.5–1), and the inhibitory–excitatory ratio k_n (0.2–1), generating a wide range of firing frequencies.

Plotting f_{cond} against f_{pred} (Fig. S2A) showed that f_{pred} systematically overestimated f_{cond}, as many points lay above the unity-slope line (dotted). Incorporating the conductance correction substantially improved the correspondence (Fig. S2B).

Generation of bimodal tuning curves

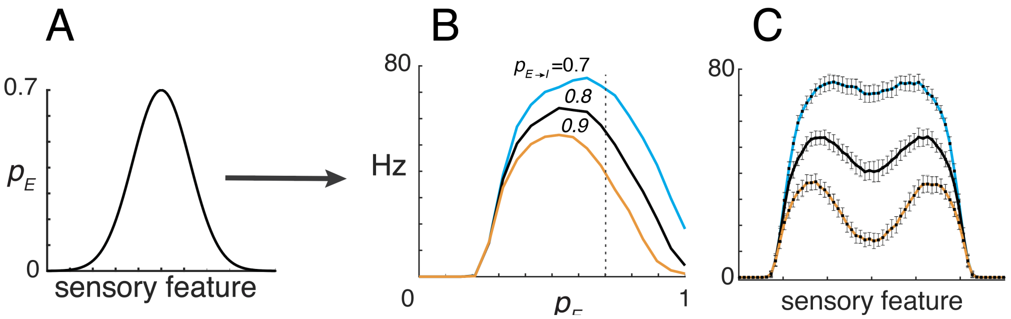

Fig S3. Effects of large tuned input. **A**, tuned input with peak of 0.7. **B**, I-O curves obtained when $p_E p_{E\to I}$ increased with p_E set at 0.7, 0.8, and 0.9. **C**, tuned responses showed a dip at the center.

In Fig. 4D of the main text, simulations were performed with a tuning curve of small amplitude (peak = 0.35) while holding $p_{E\to I}$ fixed, so that $\pi_{E\to I} = p_E p_{E\to I}$ scaled linearly with p_E. With this amplitude, responses fell on the rising portion of the I–O curves, yielding multiplicative gain modulation. In Fig. S3, the peak input probability was doubled to 0.7 (Fig. S3A). Because the evoked responses then fell on the decaying portion of the I–O curves (Fig. S3B), the resulting tuning curves exhibited a central dip (Fig. S3C).

Effects of conductance for brief stimuli

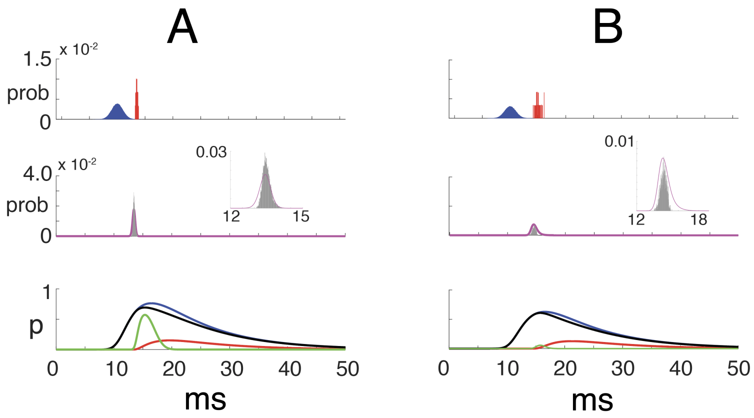

Fig S4. Predicting firing probability for transient stimuli in conductance mode. **A,top**, probability distributions of arrival times of EPSP (blue) and IPSP (red) in the reference cell. **Middle**, predicted firing probability (magenta) overlaid on the spike time histogram (gray); **Inset**, magnified view. **Bottom**, superimposed traces of $p_E\,\tilde{p}_E(t)$ (blue), $p_I\,\tilde{p}_I(t \mid \text{stim})$ (red), net input $\tilde{p}_{\text{net}}(t)$ (black), and threshold-crossing probability (green). $p_E = 0.95$, $k_n = 0.2, k_a = 1, k_{EI} = 1$. **B**, same but with $p_E = 0.75$. **Model parameters:** $n_E = 100$, $n_I = k_n\,n_E = 20$, $g_E = 0.147$ nS, $g_I = 1.045$ nS, Histograms compiled from 5,000 trials with Bin width = 0.01 ms

Simulations with brief stimuli and synaptic conductances

The simulations conducted in the main text with transient stimuli assumed linear summation of synaptic inputs. However, synaptic integration can be sublinear in some regimes, for example when synapses are spatially clustered or when inhibition produces significant shunting. To assess whether the model's predictions are robust under these conditions, simulations were repeated using conductance-based synaptic inputs (see Methods). Figure S4 shows that the results were qualitatively similar to those obtained with linear summation (compare with Fig. 6 in the main text). The effects of conductance did not qualitatively alter the predicted firing patterns, in part because action potentials occurred primarily on the rising phase of $p_E\,\tilde{p}_E(t)$ (compare the middle and bottom traces), before substantial temporal overlap with $p_I\,\tilde{p}_I(t)$.
