# Supplementary material for: Computing the effects of excitatory-inhibitory balance on neuronal input-output properties
Source: PLoS Comput Biol. 2026 Mar 9;22(3):e1013958. doi: 10.1371/journal.pcbi.1013958 (PMC12998957; doi:10.1371/journal.pcbi.1013958)
Supplement: Attachment — Submitted filename: plos_cb_review_25-07-07.pdf [file pcbi.1013958.s007.pdf]

In the manuscript, the author suggests that the response properties of spiking neurons, particularly those in the cortex, can be described by a simple model based on a probabilistic combination of excitatory and inhibitory synaptic inputs. The author considers this model in the context of feedforward inhibition, in which a reference neuron receives excitatory input from a population of input neurons. Simultaneously, this population drives a population of inhibitory interneurons located in the same circuit as the reference neuron. The reference neuron receives inhibitory input from these interneurons, which balances the excitatory input. As the interneurons are also stimulated by the excitatory input, the activity of the interneurons can co-vary with the excitatory input. This can be important for the response properties of the reference neuron, i.e., its output activity as a function of excitatory input.

The author proposes a simple model of the interaction between excitation (E) and inhibition (I), in which the synaptic inputs to the reference neuron are probabilistically combined. The model states that input activity (spikes from presynaptic neurons) only leads to a postsynaptic current in the reference neuron with a certain probability. When E and I inputs are balanced on average, a positive fluctuation of E and/or a negative fluctuation of I is necessary for the reference neuron to generate a response spike. The results start with a simple toy model, where an E input leads to a response spike, but a simultaneous I input suppresses the response by canceling or deleting the synaptic E input. This leads to a multiplicative interaction of probabilities: p_f = p_E (1 − p_I), the probability of an E input and the absence of an I input (assuming independence).

If p_E is the probability of a postsynaptic excitatory current given input spikes from the E population and p_I is the probability of a postsynaptic inhibitory current given input spikes from the I interneuron population, the author suggests by analogy with the multiplicative interaction in the toy model that the probability of a postsynaptic current is p_E,net = p_E(1-k p_I), where k is a constant. The idea is that combination of E and I inputs in the reference neuron can be described by an effective probability of postsynaptic excitation. This means that the probability of a single postsynaptic current is reduced by inhibitory inputs. If I understand correctly, the idea is that this effective probability can describe the response characteristics of the reference neuron, particularly how a multiplicative interaction of E and I probabilities affects the response.

Unfortunately, I cannot understand how this formula comes about. From a probabilistic point of view the formula states that if a single postsynaptic current occurs at a given E input spike, then it is deleted by inhibitory input with probability k p_I. In the LIF neuron model under consideration, synaptic inputs (postsynaptic currents) superpose linearly, as also noted in the manuscript. Additionally, the amplitude of individual synaptic inputs is small compared to the firing threshold; therefore, a certain number of E inputs arriving more or less simultaneously are required to generate a response spike. For simplicity, consider a short time interval (or transient synchronous input, as discussed in the Appendix, "Calculating Firing Probability with Transient Input," on page 25), during which the neuron receives input from 100 E neurons. Each neuron leads to a postsynaptic current with probability p_E. The number of synaptic inputs is then binomially distributed Bin(n_E, p_E). If they all have the same amplitude, the probability of a response spike in the absence of inhibition equals the probability that

this number is equal to or greater than the firing threshold divided by the amplitude. Now, if the neuron receives input simultaneously from n_I = 20 interneurons, each generating an additional inhibitory postsynaptic current with probability p_I, the number of inhibitory synaptic inputs is binomially distributed, Bin(n_I, p_I). If these inhibitory inputs have the same amplitude as the excitatory inputs but with a negative sign, the effective (net) number of inputs is the difference between two binomially distributed random variables. The effective number can be negative, but this does not affect the neuron's response behavior. Up to this point, the interaction between E and I inputs is purely additive. In particular, the mean of the difference is given by the difference of the means, and the variance of the difference the sum of the variances (assuming independence). The response probability is the probability that the difference between the number of E and I inputs is greater than or equal to the threshold. In this case, it can be calculated using the distribution of the difference between two binomially distributed random variables. At this point, a multiplicative interaction emerges: depending on the number of I inputs, the number of E inputs must be larger. However, I do not see how this situation can be described by a reduced multiplicative probability of the form p_E,net = p_E(1-k p_I) for the occurrence of individual excitatory inputs.

Perhaps I have overlooked something in the argument in the manuscript and would be happy to be convinced otherwise. My difficulty understanding the description through p_net(t) suggests that the manuscript may be unclear in this respect. As this description is the main conceptual and theoretical result of the manuscript, validity and understanding the derivation is important to assess whether the manuscript should be published in PLOS CB. In my opinion, the presentation and mathematical derivations are not ready yet.

The manuscript investigates the response behavior of the reference neuron in different scenarios using LIF neuron simulations and probabilistically generated inputs. The probability p_E(t) varies over time. The neuron's response behavior is characterized by its time-dependent firing rate (PSTH, averaged over independent trials). Feedforward inhibition is modeled either directly by the probability p_I(t), where a reference firing rate must be assumed for the I neurons, or by a population of interneurons that receives probabilistically generated input with probability p_E->I(t). In the various scenarios, the reference neuron exhibits different response behavior as a function of p_E and time. Depending on how p_I(t) co-varies with p_E(t), the feedforward circuit exhibits multiplicative or additive modulation and different temporal response behavior, as observed in experiments.

These simulation results are interesting and comprehensible, except for a few details (see next paragraph). One strength of the study is that it demonstrates how a simple model, in which synaptic E inputs are probabilistically combined with I inputs through feedforward inhibition, can describe and explain various phenomena observed in the response behavior of cortical neurons. The description by p_E,net (t) is not necessary for this model, and the simulation results are relevant. Of course, it would be helpful to find a compact, effective description, as attempted in the manuscript via p_E,net. One possibility would be to consider the mean and variance of the superimposed synaptic E and I inputs, i.e., the difference discussed above. These quantities could provide a

proxy for the response probability. The interesting simulation results, perhaps with more details, together with a mechanistic theoretical understanding, would make this study suitable for PLOS CB, in my view.

From a biological point of view, an important criterion for PLOS CB, the manuscript did not clarify what is physiologically modeled through the probability p_E(t). Is it unreliable, random synaptic transmission of input spikes? As in references [1-4] below? This is an important aspect of the stochasticity of neural activity dynamics in neural circuits that has not been studied often in model studies. This would be an interesting topic to explore. It is somewhat unclear whether each spike of the E-input population independently leads to a postsynaptic current in the reference neuron with probability p_E(t), which for Poisson inputs is equivalent to a time-dependent rate p_E(t) r_E n_E. If p_E(t) models unreliable, stochastic synapses, then what could be the physiological cause of the time-dependent change in probability p_E(t)? The meaning of p_I for the inhibitory interneurons seems to vary depending on the situation. In the case of longer stimuli, p_I(t) should modulate a reference rate, r_I, of the I neurons. However, it remains unclear how this rate is obtained. Is it the spike rate obtained when p_E->I = 1? I understand that p_I is determined by adjusting p_E->I. It would be clearer if it were formulated directly with rates, at least for the case of longer stimuli. For transient stimuli, p_E also seems to describe the probability that a neuron in the E-input population will emit a spike (see references [5,6] for stochastic approaches to the transmission of synchronous input activity). However, this is not entirely consistent with the independent probability, p_E->I, of input spikes to interneurons. It would be helpful to describe the assumptions and physiological significance of the model in more detail. Perhaps the author could discuss whether both interpretations—stochastic spiking and stochastic synaptic transmission—are possible.

Minor points:

1. The mathematical notation could be more precise. For example, in Eq. (2) on page 4, i_net(t) is defined in terms of p_net(t). As I understand, p_net(t) is supposed to be a deterministic quantity that describes the effective net probability of excitatory synaptic inputs. However, in Eq. (4) on page 5 i_net is used as random quantity to compute a probability, in fact there it is bar{i}_net, which is defined as the average. The latter would be the formula with p_net(t), I believe.

2. Transient stimuli: Is the value sigma_t = 0.1ms on page 15 correct? This seems almost perfectly synchronized and does not agree with the plots in Figure 6. It would be interesting to investigate the effect of feedforward inhibition on temporally more widely distributed inputs. In this context, there seems to be some relevant work on the effect of feedforward inhibition in the context of synfire-chain transmission, for instance, Ref. [7].

3. In general, there seem to be a few typos in the formulas, for example, p_EtoI instead of an arrow and \mu(t) and u(t) interchanged on page 26 (Appendix). Of course, these can be easily fixed.

References:

[1] Tsodyks, M. V., & Sejnowski, T. (1995). Rapid state switching in balanced cortical network models. Network: Computation in Neural Systems, 6(2), 111.

[2] Anthony Zador (1998). Impact of Synaptic Unreliability on the Information Transmitted by Spiking Neurons. Journal of Neurophysiology 79:3, 1219-1229.

[3] Moreno-Bote R. (2014) Poisson-Like Spiking in Circuits with Probabilistic Synapses. PLoS Comput Biol 10(7): e1003522. https://doi.org/10.1371/journal.pcbi.1003522

[4] Michael J. Leone, Chengcheng Huang, Brent Doiron: Noise Correlations in Balanced Networks with Unreliable Synapses. bioRxiv 2025.01.09.632185; doi: https://doi.org/10.1101/2025.01.09.632

[5] Burkitt, A. N., & Clark, G. M. (1999). Analysis of integrate-and-fire neurons: synchronization of synaptic input and spike output. Neural computation, 11(4), 871-901.

[6] Gewaltig, M. O., Diesmann, M., & Aertsen, A. (2001). Propagation of cortical synfire activity: survival probability in single trials and stability in the mean. Neural networks, 14(6-7), 657-673.

[7] Shinozaki, T., Okada, M., Reyes, A. D., & Câteau, H. (2010). Flexible traffic control of the synfire-mode transmission by inhibitory modulation: nonlinear noise reduction. Physical Review E—Statistical, Nonlinear, and Soft Matter Physics, 81(1), 011913.
