# Supplementary material for: Computing the effects of excitatory-inhibitory balance on neuronal input-output properties
Source: PLoS Comput Biol. 2026 Mar 9;22(3):e1013958. doi: 10.1371/journal.pcbi.1013958 (PMC12998957; doi:10.1371/journal.pcbi.1013958)
Supplement: Attachment — Submitted filename: respToRefs.pdf [file pcbi.1013958.s008.pdf]

I thank the referees for their thorough evaluation of the manuscript and for their constructive comments and suggestions. A key concern shared by referees 1 and 2 was that the earlier version did not clearly define the model variables or specify which parameters were varied in the simulations. In the revised manuscript, I have redefined several variables to provide clearer physiological interpretation, added two tables in the Results and Methods sections summarizing all parameters, and extensively revised the Appendix to make the derivations more transparent and easier to follow. Apart from these clarifications and minor figure edits, the data, analyses, and overall conclusions remain unchanged from the previous version.

Most of the substantive revisions to the main text appear in the sections Sustained Stimuli (**lines 116–192** of the revised ***marked*** manuscript) and Transient firing regime (**lines 346-358**). I describe the model more clearly, outlining the steps of the simulations in greater detail. A new table and an updated Fig. 2A now define the variables at each stage of the feedforward network. The probabilistic terms are also defined more precisely, with physiologically meaningful variables that clarify the sources of variability during stimulation. For example, I introduced explicit probability terms for synaptic efficacy between pre- and postsynaptic neurons ($p_{E\to R}$, $p_{E\to I}$, $p_{I\to R}$) and made more explicit the factors that influence inhibitory input probability. Finally, the notation has been simplified and made consistent throughout: neuronal inputs are now expressed in terms of average current, eliminating the time-dependent notation that caused confusion in the previous version (**Eqs. 2–4, lines 153–177**).

Responses to Referee 1

I am grateful to the referee for his careful reading of the manuscript and helpful comments. I have addressed all of his concerns below.

1. Referee's comment: *There is a confusing mixture of Results and Methods (model) in the current manuscript. It is not possible to understand the Results in any detail without first having a clear explanation of the model. This partly explains why the first part of the Results section contains large amounts of text that rightly belongs in the Methods. However, this leads to considerable confusion for the reader, which could be overcome by a clear description of the Methods before the Results are presented.*

Author's response: In the revised manuscript, the modeling framework is now presented in greater detail (**lines 117–110** and **Fig. 2 caption**), with an explicit description of the steps involved in calculating the neuronal inputs and responses. The equations have been reformulated and simplified by expressing inputs as average currents, thereby avoiding the time-dependent notation used in the previous version. Most of the derivations have been moved to the Supplementary Information and Methods, leaving only the key equations (Eqs. 2–4) that are essential for understanding how the manipulations in Figs. 3 and 4 affect firing. Finally, the variables are now defined more precisely, with clearer physiological interpretation (**lines 117–123**).

2. Referee's comment: *Throughout, there needs to be a clear notational difference between the average, instantaneous, and integral values of parameters, which otherwise leads to possible confusion.*

Author's response: I agree that the previous version of the manuscript used overlapping notation for different probability measures (e.g., p_E, $p_E(t)$, $\bar{p}_E$, p_I, $p_I(t)$, $\bar{p}_I$, $\hat{p}_I$, p_{net}, $p_{\text{net}}(t)$, $\bar{p}_{\text{net}}$), which led to confusion between instantaneous, average, and integral quantities. In the revised version, I have

made these distinctions explicit and consistent throughout the text and figures. Specifically, p_E now denotes the probability that a stimulus evokes firing in an excitatory afferent, and p_I represents the effective probability that an inhibitory afferent to the reference neuron becomes active (i.e., the probability that an inhibitory spike reaches the reference cell). The term p_I is defined as the product of factors related to neuron number, synaptic strength, and the probability that an inhibitory neuron fires (Eq. 3). These quantities are used to scale the average synaptic currents (Eq. 2) and the probabilities associated with transient inputs (Eqs. 6, 7). The time-dependent quantities $p_E(t)$, $p_I(t)$, and $p_{\text{net}}(t)$ are introduced only in the section on temporal firing profiles (Fig. 5), where they describe instantaneous probabilities.

Other notations (e.g., integrated or empirically averaged forms) are used only for derivations and have been moved to the Supplementary Material to avoid cluttering the main text. In addition, the input–output curves in Figs. 3 and 4 are now plotted as functions of p_E, rather than $\bar{p}_E$, to maintain consistency. Together, these changes ensure a clear and consistent notational distinction between instantaneous, average, and integral quantities across the manuscript.

3. Referee's comments: *Figure 2: The caption is missing the details for subplots B(ii) and B(iii). It is also unclear what the bin-width is for the spike histograms. Some of the described steady-state values in the caption do not agree with those plotted.*

Author's response: Thank you for flagging these errors. They have been rectified in the figure legends. Bin width was 0.01 ms and number of sweeps was 10000. The values of the probabilities and steady-state values are now correctly stated.

4. Referee's comments: *Appendix: The Equation-numbers and Figure-numbers in the appendices need to be distinguished from those in the main text (this is most simply done by adding an "S" at the start of each number). Otherwise, there is confusion about which equation or figure is being referred to.*

Author's response: I've re-labeled the equations and figures in the Supplementary material with an "S" to avoid confusion with those in the main text.

5. Referee's comments: *Appendix: The Figures in the appendix should be given subplot labels similar to that given to subplots in the main text.*

Author's response: I have added the subplot labels to the figures.

6. Referee's comments: *Appendix: "Correcting for conductance" and Figure S1: Equation(S1) needs more information: what is "coul" and "sec"? How is G_E defined? Confusing notation to have $g_E(t)$ and $g_E(v)$ – one of these needs to be different (i.e., $\hat{g}_E(v)$). How is G_E defined (i.e., what are the limits on the time-integral)?*

Author's response: I have re-written the derivation so that is clearer (**p. 7-8** of supplementary information). I removed the terms G_E, G_I and simplified the notations. I also added the limits of integration in the integrals (eq. S33) The notation for conductance is actually $g_E(t)$ while that for charge is $q_E(t)$. The problems is that "g's" and "q's" look similar in Latex. Rather than re-defining them throughout the manuscript, I added a clarifying sentence and wrote the symbols in text to emphasize the difference and also included the units (**p7, eqs. S33 and S34** of supplementary information),

7. Referee's comments: *Appendix: Effects of conductance for brief stimuli" & Figure S3: Details of the parameters are currently missing. The parameter details need to be sufficient for the plots to be reproduced. ?*
Author's response: I included the details of the simulation in the figure caption (re-labeled as Fig. S4 in revised manuscript). They were identical to those in Fig. 6 of the main text except the synaptic inputs were delivered as conductances. To maintain equal EPSP and IPSP amplitude at resting potential, the excitatory and inhibitory conductances were set to $g_E = 0.147nS$ and $g_I = 1.045$ nS, respectively.

8. Referee's comments: *One omission is a discussion of the role of spontaneous activity, which impacts the latency-to-first-spike in IAF neurons – the lack of spontaneous activity means that the voltage starts at the reset value for transient stimuli.*

Author's response: I thank the referee for pointing out this important limitation. Background activity can indeed influence both transient and sustained firing. We are currently exploring how to incorporate probabilistic contributions from sources outside the feedforward network, although this has proven to be more complex than initially anticipated. To acknowledge this issue, I have added the following paragraph to the Discussion (**lines 494–499**):

"Fourth, spontaneous activity—whether intrinsically generated or originating from neurons outside the feedforward pathway—is not explicitly included in the present analysis. Background activity can alter the initial conditions of the network, influence transient responses [1, 2], and contribute to steady-state firing. In principle, such effects could be incorporated into the probabilistic framework if expressed in terms of effective input probabilities, although this would require additional theoretical development."

9. Referee's comments: *one omission appears to be the substantial body of related work on EI-balanced networks by Deneve and colleagues (refs below), which seems to be very relevant to the current study*

Author's response: I thank the referee for recommending potentially relevant studies of Boerlin et al., Brendel et al, Gutierrez and Deneve, and Gutkin et al. These studies primarily investigate excitation–inhibition balance in recurrent networks. Boerlin et al. formulated a predictive coding framework in which irregular spiking and E–I balance emerge from neurons minimizing a population-level prediction error. Brendel et al. demonstrated how efficient spike-by-spike coding can be learned through local plasticity rules that maintain E–I balance, while Gutierrez and Deneve analyzed how adaptation in balanced recurrent networks resolves cost–accuracy tradeoffs to stabilize population codes. Gutkin et al. provided a theoretical analysis of how excitatory–inhibitory coupling generates oscillations, synchrony, and stability in recurrent circuits. In contrast, the present study focuses on *feedforward inhibitory circuits* and introduces a *probabilistic framework* describing how excitatory inputs are either transmitted or canceled by inhibition. Thus, while all these studies address E–I interactions at a broad level, the modeling approaches, network architectures, and scientific aims are distinct and complementary.

Responses to Referee 2

I am very grateful to the referee for the detailed and thoughtful critique. I agree that the description of the terms required clarification, and I have addressed these points as outlined below.

1. Referee's comment: *For simplicity, consider a short time interval, during which the neuron receives input from 100 E neurons. Each neuron leads to a postsynaptic current with probability p_E. The number of synaptic inputs is then binomially distributed Bin(n_E, p_E). If they all have the same amplitude, the probability of a response spike in the absence of inhibition equals the probability that this number is equal to or greater than the firing threshold divided by the amplitude. Now, if the neuron receives input simultaneously from n_I = 20 interneurons, each generating an additional inhibitory postsynaptic current with probability p_I, the number of inhibitory synaptic inputs is binomially distributed, Bin(n_I, p_I). If these inhibitory inputs have the same amplitude as the excitatory inputs but with a negative sign, the effective (net) number of inputs is the difference between two binomially distributed random variables. The effective number can be negative, but this does not affect the neuron's response behavior. Up to this point, the interaction between E and I inputs is purely additive. In particular, the mean of the difference is given by the difference of the means, and the variance of the difference the sum of the variances (assuming independence). The response probability is the probability that the difference between the number of E and I inputs is greater than or equal to the threshold. In this case, it can be calculated using the distribution of the difference between two binomially distributed random variables. At this point, a multiplicative interaction emerges: depending on the number of I inputs, the number of E inputs must be larger....*

....However, I do not see how this situation can be described by a reduced multiplicative probability of the form p_E,net = p_E(1-k p_I) for the occurrence of individual excitatory inputs.....My difficulty understanding the description through p_net(t) suggests that the manuscript may be unclear in this respect.

The description by $p_{E,net}(t)$ is not necessary for this model, and the simulation results are relevant. Of course, it would be helpful to find a compact, effective description, as attempted in the manuscript via $p_{E,net}(t)$.

Author's response: I agree with the referee that the model can, in principle, be expressed entirely in terms of independent excitatory and inhibitory inputs combined additively, and that the simulations themselves do not require the explicit introduction of p_{net}. The formulation as a difference of two binomially distributed random variables is indeed a valid description of the underlying process.

However, the motivation for introducing p_{net} was not computational necessity but to obtain a clearer and more tractable formulation that is consistent with a feedforward inhibitory circuit. Because inhibition is recruited by the same afferents that drive excitation, the interaction can be naturally interpreted as a cancellation or survival probability for excitatory events, rather than as the subtraction of two independent stochastic variables. The multiplicative form

$$p_{\text{net}} = p_E(1 - p_I)$$

thus represents the expected fraction of excitatory inputs that survive feedforward inhibition, providing a bounded and physiologically interpretable measure of effective excitation.

This probabilistic formulation is consistent with the additive framework: the mean net input current remains proportional to the difference of the excitatory and inhibitory means but is expressed in a bounded, non-negative form. The survival-probability view avoids the need to impose *ad hoc* truncations on negative values that can arise from the difference-of-binomials expression, while still capturing the same dependence on p_E and p_I.

The compact p_{net} formulation also facilitates analytical derivations—for example, of the mean net current (Eq. 4 of revised ***marked*** manuscript) and input–output relationships—while automatically incorporating parameters such as interneuron recruitment and synaptic reliability that determine p_I. Thus, although not required for the simulations, p_{net} serves as a concise and physiologically interpretable formulation that complements the classical additive framework.

To clarify these points, the revised text (**lines 171–177** of revised ***marked*** manuscript) now explicitly contrasts the current probabilistic form with classical add–subtract models: *"This compact probabilistic form incorporates interneuron recruitment and synaptic reliability, and provides an effective representation of the mean net excitatory drive under feedforward coupling. In contrast to classical add–subtract models (e.g., for Poisson processes [51]), in which excitation and inhibition are treated as independent and subtracted directly, the present formulation yields a probabilistic measure that, by construction, remains within a normalized range (approximately [0, 1]) for realistic parameter values, thereby avoiding unbounded subtraction of independent terms."* These distinctions are also summarized in the Discussion (**lines 410–416**).

Finally, in **Fig. 2B (top)**, I now show the net current (and corresponding probability, right axis) obtained by direct subtraction of excitatory and inhibitory currents (gray) alongside that derived under conditional inhibition (black), to emphasize the conceptual and quantitative differences between the two formulations.

2. Referee's comment *From a biological point of view, an important criterion for PLOS CB, the manuscript did not clarify what is physiologically modeled through the probability p_E(t). Is it unreliable, random synaptic transmission of input spikes? ..... It is somewhat unclear whether each spike of the E-input population independently leads to a postsynaptic current in the reference neuron with probability p_E(t), which for Poisson inputs is equivalent to a time-dependent rate p_E(t) r_E n_E. If p_E(t) models unreliable, stochastic synapses, then what could be the physiological cause of the time-dependent change in probability p_E(t)?*

Author's response: In the revised manuscript, the probabilities associated with the feedforward inhibitory network are now explicitly defined in Table 1 and illustrated in Fig. 2A. The new formulation separates the sources of variability during stimulation. Specifically, $p_E(t)$ represents the probability that an excitatory afferent neuron fires at time t in response to the stimulus—that is, the time-varying likelihood of presynaptic spiking across the excitatory population. It does *not* model stochastic or unreliable synaptic transmission. The reliability of synapses is instead expressed by the probabilities that an afferent spike evokes a postsynaptic current (EPSP or EPSC), given by $p_{E\to R}$ for the reference neuron and $p_{E\to I}$ for each inhibitory neuron. Accordingly, the probability of an excitatory input occurring in the reference and inhibitory neurons is given by $p_E(t)p_{E\to R}$ and $p_E(t)p_{E\to I}$, respectively (Eq. 4, time-independent form)..

The time dependence of $p_E(t)$ arises from the temporal structure of the stimulus itself: changes in stimulus amplitude or duration modulate the instantaneous firing probability of the afferent population, which in turn determines the time-varying synaptic drive to both excitatory and inhibitory neurons. To maintain consistency and simplify notation, as suggested by Referee 1, the time-dependent form is used only in the description of Fig. 5 and the transient-stimulus condition in the revised manuscript.

3. Referee's comment: *One possibility would be to consider the mean and variance of the superimposed synaptic E and I inputs, i.e., the difference discussed above. These quantities could provide a proxy for the response probability.*

Author's response: I appreciate the referee's suggestion to approximate the response probability using the mean and variance of the summed excitatory and inhibitory inputs. While feasible in principle, this approach provides limited insight into how physiological factors such as network size, synaptic reliability, or recruitment contribute to the response. These parameters influence the mean and variance in complex, nonlinear ways. By contrast, the p_{net} formulation expresses these dependencies explicitly and remains bounded and interpretable as the fraction of excitatory events that survive inhibition. Thus, although a mean–variance description could be derived, it would be less obvious how circuit parameters shape the probability of firing.

4. Referee's comments: *The meaning of p_I for the inhibitory interneurons seems to vary depending on the situation. In the case of longer stimuli, p_I(t) should modulate a reference rate, r_I, of the I neurons. However, it remains unclear how this rate is obtained. Is it the spike rate obtained when p_E-¿I = 1? I understand that p_I is determined by adjusting p_E-¿I. It would be clearer if it were formulated directly with rates, at least for the case of longer stimuli.*

Author's response: I thank the referee for raising this important point. In the revised manuscript, I clarify that p_I represents the effective probability that an inhibitory input arrives at the reference neuron. This term incorporates several factors, including the probability that inhibitory neurons fire (see below), whereas r_I denotes the mean firing rate of the inhibitory population once activated (**Table 1, lines 163–168**). The average excitatory synaptic input to the inhibitory cell is given by (Eq. 2):

$$\bar{i}_{E\to I} = p_E\, p_{E\to I}\, n_E\, q_E\, r_E,$$

where $p_E p_{E\to I}$ gives the probability that a synaptic input will appear in the inhibitory neuron during stimulation. If this excitatory current exceeds rheobase, the inhibitory neuron fires at a rate r_I, which corresponds to the mean firing rate under full excitatory recruitment (i.e., when $p_{E\to I} = 1$). Hence, the effective inhibitory rate can be modulated by changing either p_E or $p_{E\to I}$.

The average inhibitory input to the reference cell (unconditioned by p_E) is then:

$$\begin{aligned}\bar{i}_{I\to R} &= \hat{p}_I\, p_{I\to R}\, n_I\, q_I\, r_I \\ &= p_I\, p_{E\to R}\, n_E\, q_E\, r_E,\end{aligned}$$

where $\hat{p}_I$ is the probability that the inhibitory neuron fires. For analytical convenience, p_I is defined as the *effective inhibitory probability*, which incorporates $\hat{p}_I$ together with constants that relate inhibitory parameters to the excitatory reference population (Eq. 3):

$$p_I = \hat{p}_I\, k_n\, k_q\, k_r\, k_{EI}, \quad \text{with } k_{EI} = \frac{p_{I\to R}}{p_{E\to R}}.$$

This formulation expresses inhibitory drive in the same normalized units as excitation, allowing direct comparison between excitatory and inhibitory contributions.

For short, transient stimuli, the time-varying inhibitory probability is given by $p_I \tilde{p}_I(t)$, where $\tilde{p}_I(t)$ follows the temporal profile of the compound inhibitory input (**lines 347–351**) and represents the instantaneous probability that an inhibitory event occurs at time t.

5. Referee's comments: *For transient stimuli, p_E also seems to describe the probability that a neuron in the E-input population will emit a spike (see references [5,6] for stochastic approaches to the transmission of synchronous input activity). However, this is not entirely consistent with the independent probability, p_E-¿I, of input spikes to interneurons. It would be helpful to describe the*

assumptions and physiological significance of the model in more detail. Perhaps the author could discuss whether both interpretations—stochastic spiking and stochastic synaptic transmission—are possible.

Author's response:

The referee is correct that, in principle, p_E could be interpreted either as stochastic presynaptic spiking or as stochastic synaptic transmission. As stated above, the revised manuscript now distinguishes the two sources of variability: p_E specifically denotes the probability that an excitatory afferent fires in response to the stimulus, whereas synaptic reliability is represented separately by $p_{E\to R}$ and $p_{E\to I}$. These two factors together determine the overall probability that a postsynaptic event occurs in each target neuron ($p_E p_{E\to R}$ and $p_E p_{E\to I}$, respectively), thereby preserving a clear separation between presynaptic firing variability and synaptic reliability. This distinction clarifies the physiological assumptions of the model and ensures consistency with stochastic spiking frameworks.

6. Referee's comments: *The mathematical notation could be more precise. For example, in Eq. (2) on page 4, $i_{net}(t)$ is defined in terms of $p_{net}(t)$. As I understand, $p_{net}(t)$ is supposed to be a deterministic quantity that describes the effective net probability of excitatory synaptic inputs. However, in Eq. (4) on page 5 i_{net} is used as random quantity to compute a probability, in fact there it is $\bar{i}_{net}(t)$, which is defined as the average. The latter would be the formula with $p_{net}(t)$, I believe.*

Authors response: I thank the referee for pointing out the inconsistencies in the equations. To avoid confusion, I now clarify that $i_{\text{net}}(t)$ denotes the instantaneous (stochastic) synaptic current, whereas its mean value, $\bar{i}_{\text{net}} = \mathbb{E}[i_{\text{net}}(t)]$, is determined by the deterministic factor $p_{\text{net}}(t)$. Accordingly, the average net current is defined as (Eq. 4, see also **p. 4** in supplementary information):

$$\bar{i}_{\text{net}} = n_E q_E r_E p_{E\to R}\, p_{\text{net}}.$$

The time-varying form of this expression is presented in the section on temporal firing profiles (Fig. 5).

Equation 4 of the previous manuscript (eq. 5 of revised manuscript) has been rewritten as:

$$r_{\text{fluct}} = \Pr\big(n_{\text{net}} > n_\theta\big)\, r_{rh},$$

where n_{net} is the stochastic net number of excitatory inputs and n_θ is the minimum number needed to reach threshold.

7. Referee's comments: *Transient stimuli: Is the value sigma_t = 0.1ms on page 15 correct? This seems almost perfectly synchronized and does not agree with the plots in Figure 6.*

Authors' response: Thank you for pointing that out. The actual value is 1 ms, which is corrected in the present version. The corrected value is now in **eq. 10** (page 19) of the revised manuscript.

8. Referee's comments: *It would be interesting to investigate the effect of feedforward inhibition on temporally more widely distributed inputs. In this context, there seems to be some relevant work on the effect of feedforward inhibition in the context of synfire-chain transmission, for instance, Ref. [7].*

Authors' response:

I thank the referee for this suggestion. To assess the effect of temporally distributed inputs, I performed additional simulations with broader excitation (increased σ_t to 5 ms). As shown in the figure below, the results exhibit little qualitative difference from the narrow-input case. Preliminary analysis suggests that this occurs because broader input distributions recruit fewer inhibitory neurons—reduced synchrony makes inhibitory firing less likely—and because the inhibition that does occur arrives later on the falling phase of the compound EPSP, where it has less influence on spiking. The total number of presynaptic spikes was held constant, so as σ_t increased, the peak of the compound input decreased proportionally, resulting in fewer spikes in both the reference and inhibitory cells. While more systematic exploration would be required to quantify these effects fully, the present simulations suggest that feedforward inhibition remains most effective for synchronous or near-synchronous input volleys.

Regarding Ref. [7], the cited study examined synfire-chain transmission in which the timing of inhibition was varied independently of excitation, including cases where inhibitory input preceded excitation. In contrast, in the present model, inhibition arises through fixed feedforward connections and therefore naturally follows excitation. I also tested cases in which inhibition was artificially advanced to occur before excitation, but such manipulations are not physiologically realistic. A proper investigation of this regime would require modifying the membrane and synaptic properties of the inhibitory neurons, or the amplitude and temporal profile of the afferent input, since these factors determine the distribution of IPSP onset times.

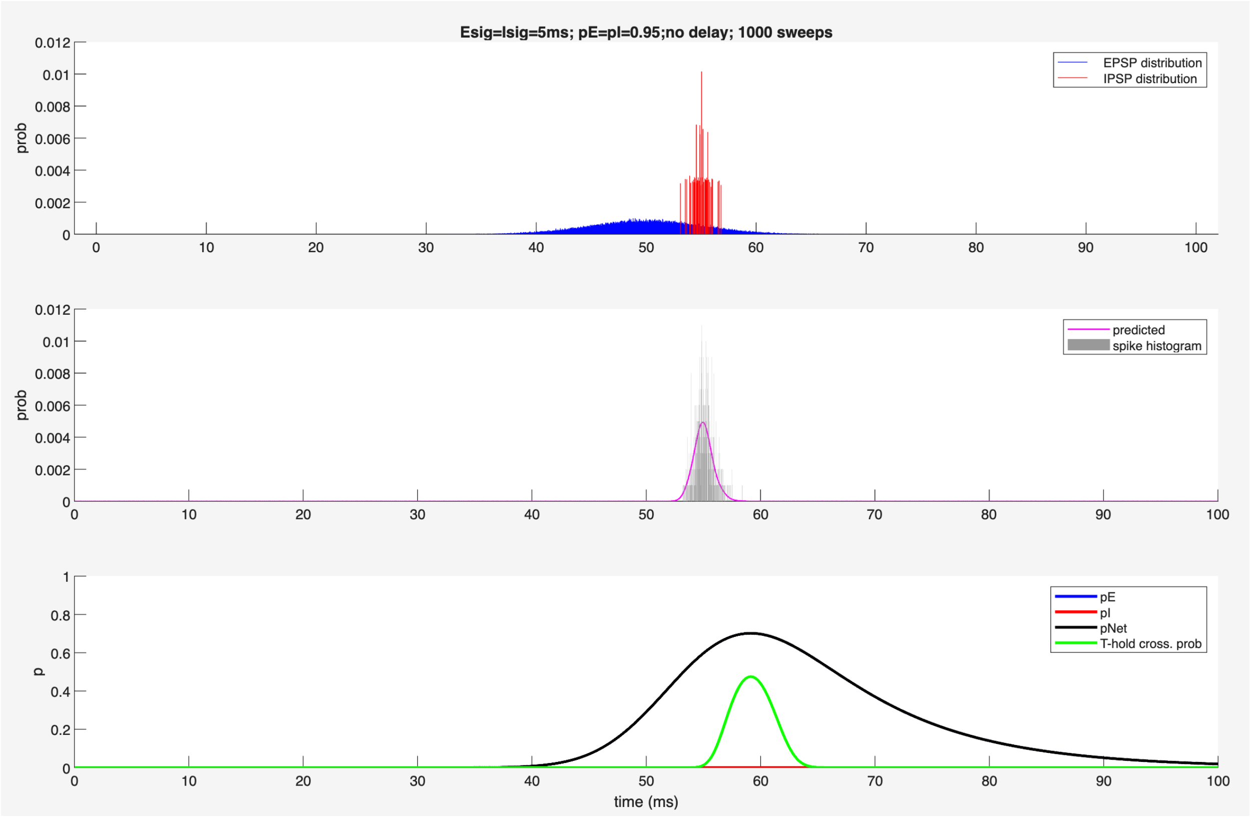

9. Referee asks if publications are relevant:

Author's response: I thank the referee for drawing my attention to these related studies. While all of

them share a broad interest in probabilistic synaptic mechanisms and excitation–inhibition balance, their modeling approaches and aims differ from the present work. Burkitt and Clark examined how stochastic synaptic input and temporal jitter affect spike timing in integrate-and-fire neurons, whereas Moreno-Bote analyzed how probabilistic synaptic transmission within recurrent cortical networks gives rise to firing variability. Tsodyks and Sejnowski similarly studied balanced recurrent networks in which irregular activity and rapid state switching emerge from probabilistic synapses. In contrast, the present study focuses on feedforward inhibitory circuits and a probabilistic framework for stimulus-evoked responses, rather than on network states or recurrent balance. For this reason, these works are conceptually related but not directly pertinent to the specific questions addressed here.

Responses to Referee 3

I thank the referee for carefully reading the manuscript and offering insightful comments and suggestions.

1. Referee's comments: *The probabilities, p_E and p_I, are probabilities per unit time or alternatively, I'm thinking of time in a discretized way. What is somehow important to clarify, I think, is that, in the model, I'm thinking of neural integration (and hence spike generation) as a memory-less process, i.e., I can determine the probability of spiking (in this time bin, or per unit time) just looking at current inputs. There are situations, that have been extensively studied (e.g., escape noise or spike-response model), where this is indeed justified; but not always. This should be somehow shortly discussed.*

Author's response: I thank the referee for pointing this out. In the short-stimulus (binwise) formulation, the model adopts a memoryless approximation, consistent with binomial and Poisson treatments in which spiking is determined by the inputs within the current bin. For brief stimuli, the firing probability at time t is defined conditional on no spike having occurred in any earlier bin, thereby implicitly incorporating past spiking history into the calculation. This approximation is justified because the stimulus duration and integration window are short compared to membrane and synaptic time constants, so the neuron's instantaneous drive dominates its spiking probability. For long-duration stimuli, the relevant variable is the average synaptic current, which depends only on the overall rate and distribution of synaptic events. In this regime, the precise temporal sequence of past inputs is not required, as the mean current is determined entirely by their statistical properties.

I added a paragraph in the Discussion that addresses your point (**lines 500-509** of revised ***marked*** manuscript):

"Finally, a potential limitation of the framework is that spiking is described as being determined by inputs within the current time bin, without explicit dependence on earlier inputs. However, this limitation is partly alleviated by the structure of the model. For brief stimuli, the probability of a spike at a given time is defined so that no spike has occurred in any earlier bin; in this way, past spiking history is implicitly incorporated into the first-spike probability. For long-duration stimuli, the focus shifts to the average synaptic current, which depends only on the overall rate and distribution of synaptic events. In this regime, the precise sequence of inputs is less critical, since the mean current is determined by their statistical properties. Thus, while the framework does not capture history-dependent phenomena such as adaptation or refractoriness, it provides a tractable and biologically interpretable description of instantaneous integration, and it can be extended in future work to incorporate explicit history effects where needed."

2. Referee's comments: *Why introducing 3 different symbols, k_n, k_q, k_r, in Eq. 2 when they all occur as a product? Also this entails, it seems to me, a problem in justifying why this product is expected to be smaller than 1 (see the discussion in Limitations, which, by the way, is not fully convincing). One could just by-pass all of this by introducing, one k, stipulate that it is smaller than 1 (by definition) and then discuss which 'real-world' variables (e.g., relative proportion of excitatory and inhibitory neurons) are supposed to affect its value and how.*

Author's response: I introduced the constants kn,kq,krk_n, k_q, k_rkn,kq,kr separately, rather than a single lumped parameter, primarily to be transparent about which biological factors contribute to the effective inhibitory probability. Each constant corresponds to a measurable ratio (e.g., number

of inhibitory to excitatory afferents, relative synaptic amplitudes, relative firing rates). Explicitly separating these factors allows one, at least in principle, to predict how a specific change (such as increasing inhibitory rate or synaptic strength) would affect firing. Because these are defined as ratios, precise absolute measurements are not required; relative values are sufficient to explore model behavior.

I also agree that the condition that the product is between 0 and 1 is not guaranteed in every system, and available data are often incomplete. In practice, one can use ballpark estimates from the literature or acknowledge that the model's applicability may be limited in certain regimes. A practical solution is to introduce a smooth saturating transform to the product to keep it within probabilistic bounds. I added text to clarify this point and to emphasize that the model is intended to be predictive only when the effective inhibitory probability remains within a biologically plausible range.

I rewrote the paragraph to incorporate the referee's concerns (**lines 477-485**):

"Second, the constants (k_n, k_{EI}, k_q, k_r) that scale p_I are chosen (see Table 1) so that their product lies between 0 and 1, ensuring that p_I can be interpreted as a probability. Although precise values for these constants are unlikely to be fully available in any given system, reasonable estimates can be drawn from different sources (k_n, [3, 4]; k_a, k_q, [5, 6, 7]; k_r,[5, 6, 7]; k_{EI},[8, 9, 10]). In addition, k_q or k_a are likely to be dynamic, reflecting differential short-term plasticity of EPSPs and IPSPs [6, 11]. For modeling purposes, the parameters should be set so that the product is unlikely to exceed 1. As a safeguard, one may also apply a smooth saturating transform, such as $p_I(t) = 1 - \exp[-k_n k_{EI} k_q k_r \, p_I(t)]$, to preserve the probabilistic interpretation of p_I."
